# Preparation and Physicochemical Properties of Vinblastine Microparticles by Supercritical Antisolvent Process

**DOI:** 10.3390/ijms131012598

**Published:** 2012-10-03

**Authors:** Xiaonan Zhang, Xiuhua Zhao, Yuangang Zu, Xiaoqiang Chen, Qi Lu, Yuliang Ma, Lei Yang

**Affiliations:** State Engineering Laboratory for Bio-Resource Eco-Utilization, Northeast Forestry University, 150040 Harbin, China; E-Mails: zyzolanew@sohu.com (X.Z.); xiuhuazhao@nefu.edu.cn (X.Z.); cxqnefu@126.com (X.C.); luqi42700473@126.com (Q.L.); mayuliang54210@163.com (Y.M); ylnefu@163.com (L.Y)

**Keywords:** vinblastine, supercritical antisolvent, micronization, physicochemical property, *Catharanthus roseus*

## Abstract

The objective of the study was to prepare vinblastine microparticles by supercritical antisolvent process using *N*-methyl-2-pyrrolidone as solvent and carbon dioxide as antisolvent and evaluate its physicochemical properties. The effects of four process variables, pressure, temperature, drug concentration and drug solution flow rate, on drug particle formation during the supercritical antisolvent process, were investigated. Particles with a mean particle size of 121 ± 5.3 nm were obtained under the optimized process conditions (precipitation temperature 60 °C, precipitation pressure 25 MPa, vinblastine concentration 2.50 mg/mL and vinblastine solution flow rate 6.7 mL/min). The vinblastine was characterized by scanning electron microscopy, X-ray diffraction, Fourier-transform infrared spectroscopy, mass spectrometry and dissolution test. It was concluded that physicochemical properties of crystalline vinblastine could be improved by physical modification, such as particle size reduction and generation of amorphous state using the supercritical antisolvent process. Furthermore, the supercritical antisolvent process was a powerful methodology for improving the physicochemical properties of vinblastine.

## 1. Introduction

*Catharanthus roseus* is a well-known medicinal tropical perennial sub-shrub belonging to the family Apocynaceae. It is regarded as a rich source of pharmaceutically important terpenoid indole alkaloids [1]. Indigenous to Madagascar, *C. roseus* is now widely distributed, and is cultivated in China, India, Indonesia, Australia, North and South America [2]. Vinblastine is the most important *C. roseus* terpenoid for the pharmaceutical industry [3]. It is currently used to treat a wide variety of neoplasms and is recommended for the treatment of Hodgkin’s disease, acute leukemia, and choriocarcinoma which is resistant to other therapies [4]. However, vinblastine has poor water solubility, in this case, its dissolution in biological liquids is also practically poor and has a low bioavailability [5]. Many approaches have been developed to improve solubility and to enhance the bioavailability of poorly soluble drugs. These include solid dispersion [6], salt formation [7], inclusion complex [8,9] and microemulsion [10]. In recent years, significant effort has been made to develop microparticles for drug delivery [11–14]. Physical modifications often aim to increase the surface area and dissolution rate of the particles, therefore, current research is focused on generation of amorphous states or particle size reduction.

Supercritical antisolvent process is an available technology to prepared drug microparticles, without the need for grinding procedure. In this process, the drug is firstly dissolved in the solvent and then the drug solution is quickly sprayed into supercritical fluid. Precipitation occurs immediately by a rapid recrystallization of the drug. In general, supercritical antisolvent process can control particle size of production, moreover the solvent can be fully recovered, is an environmentally friendly technology. In supercritical antisolvent process, operating parameters have a great influence on particle size, such as temperature, pressure, drug concentration, *etc.* [14–16].

The purpose of this study was to prepare vinblastine microparticles by supercritical antisolvent process and evaluate their physicochemical properties. The parameters of supercritical antisolvent process such as pressure, temperature, drug concentration and drug solution feed rate and how they affect the morphology and particle size of microparticles have been studied, and then a series of analytical methods have been used to characterize the samples before and after the supercritical antisolvent processing.

## 2. Results and Discussion

### 2.1. Morphology

**Figure 1:**
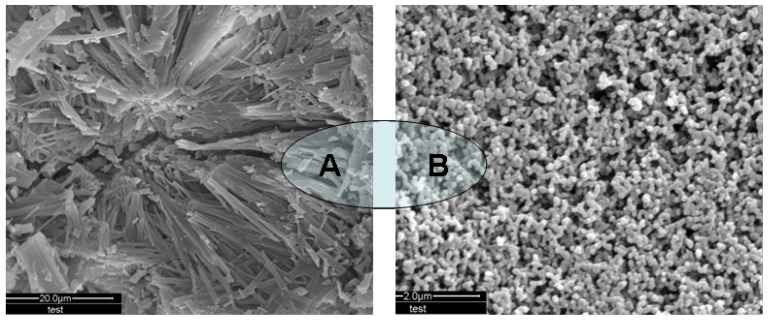
SEM images of unprocessed vinblastine and processed vinblastine. (**A**) SEM image of unprocessed vinblastine; (**B**) SEM images of processed vinblastine precipitated under optimum conditions (trial no. 10 in Table 1).

Compared with unprocessed drug, the SEM images show plenty of very small submicron particles after the supercritical antisolvent process. From Figure 1A, unprocessed vinblastine particles are irregular strip crystals, ranging in length from 1 to 20 μm. Figure 1B shows the SEM image of micronized vinblastine precipitated from *N*-methyl-2-pyrrolidone (NMP) under optimum conditions (trial no. 10 in Table 1). It is clear from the figure that processed vinblastine particles are spherical.

### 2.2. Optimum Parameters of Supercritical Antisolvent Process by L_16_ (4^5^) Orthogonal Design

**Figure 2:**
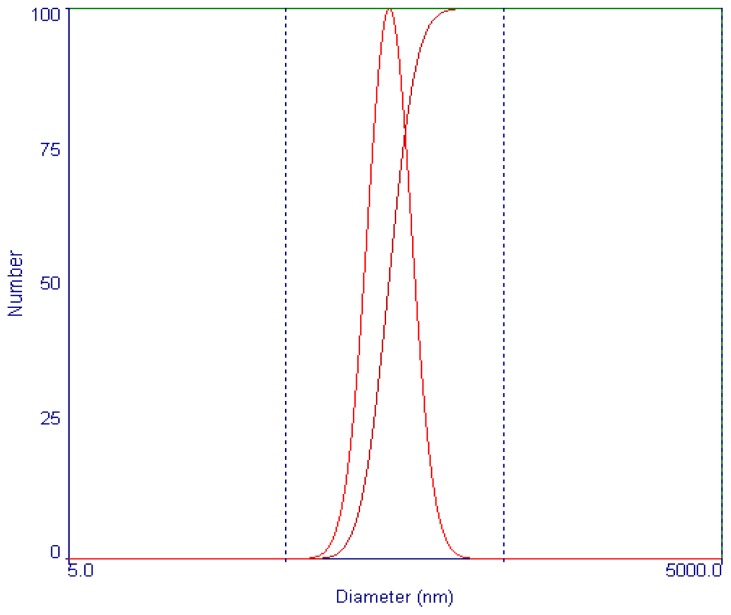
The particle size distribution of processed vinblastine.

To the best of our knowledge, various parameters play a great role in the optimization of the experimental conditions for the supercritical antisolvent process. In fact, the concentration of drug solution, precipitation temperature, precipitation pressure and drug solution flow rate are generally considered to be the most important factors. The investigated levels of each factor were selected depending on the preliminary experiment results of the single-factor. The optimization of the suitable operating conditions in supercritical antisolvent process can be carried out step by step or by using an experimental design. In the present study, all selected factors were examined using an orthogonal L_16_ (4^5^) test design. The total evaluation index was used to analyse by statistical methods. The analysis results of orthogonal test, performed by statistical software Design Expert 8.0 (Stat-Ease, Inc.: Minneapolis, MN, USA, 2010), are presented in Table 1. The results of experiments presented in Table 1 indicate that the mean particle size (MPS) of micronization vinblastine varied between 121 and 480 nm. The maximum MPS was found for trial no. 1 and the lowest for trial no. 10. The factors influence the MPS of micronization vinblastine were listed in a decreasing order as follows: C > B > D > A according to the *R* value. So, the minimum MPS of micronization vinblastine was obtained when the vinblastine concentration, drug solution flow rate, precipitation pressure and precipitation temperature were A_2_B_2_C_4_D_3_ (2.50 mg/mL, 6.7 mL/min, 25 MPa and 60 °C), respectively. Through a confirmatory test, smaller micronized vinblastine was obtained, with a MPS of 121 ± 5.3 nm. The particle size distribution of micronized vinblastine is shown in Figure 2. And the purity and yield of micronized vinblastine obtained is 98.8% and 81.2%, respectively.

The residual solvent of micronized active agent by SAS process is very low. According to literature [14], the residual DMSO content in micronized CPT by SAS method is 1244 ppm, which met ICH requirements and is suitable for pharmaceutical use. But the quantity of residual NMP of micronized vinblastine must be determined by GC method when it was applied in pharmaceutical studies.

### 2.3. Physicochemical Properties of Vinblastine Microparticles

#### 2.3.1. Fourier-Transform Infrared (FTIR) Spectroscopy Analysis

**Figure 3:**
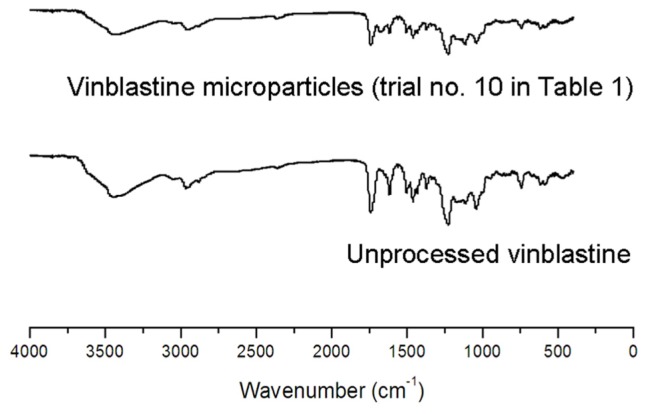
FTIR spectra of unprocessed and processed vinblastine.

We performed some analyses on unprocessed and processed vinblastine to obtain information on the change of physical and chemical structure after supercritical antisolvent process. FTIR spectra of the unprocessed and processed vinblastine (trial no. 10 in Table 1) are shown in Figure 3. The assignments of bands are as follows: 3437 cm^−1^ (O–H stretching vibrations), 2935 cm^−1^ (overlapping C–H stretching vibrations peaks of methyl, methylene and –CH), 2879 cm^−1^ (methylene C–H stretching vibrations), 1715 cm^−1^ (stretching vibration of carbonyl group). It can be seen that FTIR spectra between unprocessed and processed vinblastine do not show any significant differences.

#### 2.3.2. Mass Spectra Analysis

**Figure 4:**
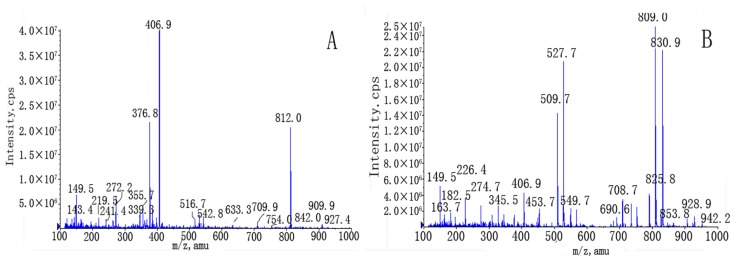
LC-MS-MS spectra of unprocessed and processed vinblastine.

Figure 4 shows MS spectra of unprocessed and processed vinblastine. It can be seen that no modification occurred in molecular weight. The two forms exhibit the same molecular weight. This explains why there were no varieties about chemistry structure of vinblastine before/after supercritical antisolvent process. Therefore, the supercritical antisolvent process has not induced chemical structure change of vinblastine.

#### 2.3.3. X-ray Powder Diffraction (XRD) Analysis

**Figure 5:**
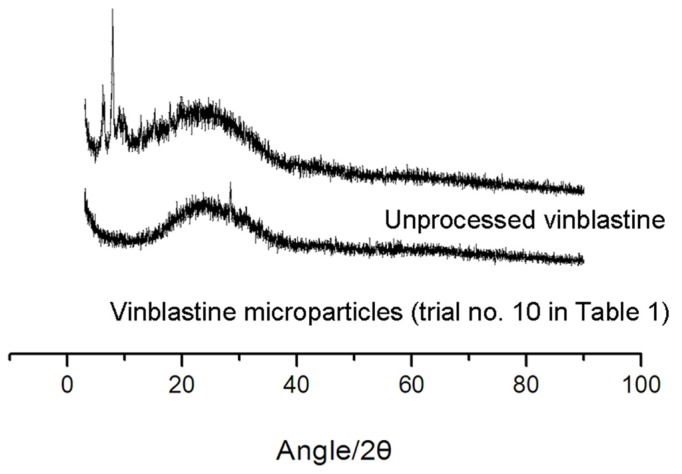
Powder X-ray diffraction patterns of vinblastine before/after SAS process.

To investigate further crystalline of particles, XRD analysis was performed. Figure 5 shows the XRD results for processed and unprocessed vinblastine particles. The presence of several distinct peaks in the XRD of unprocessed vinblastine at the diffraction angles of 2*θ* = 5.9°,13.3°,17.68°, 14.9°, 8.9°, 20.16° and 25.54° reveals the drug is present as a crystalline form. The processed vinblastine, on the other hand, presents a diffract gram with one weak peak at 25.54° of 2*θ*. This fact suggests that vinblastine particles after supercritical antisolvent process have less crystalline.

#### 2.3.4. Dissolution Studies *in Vitro*

**Figure 6:**
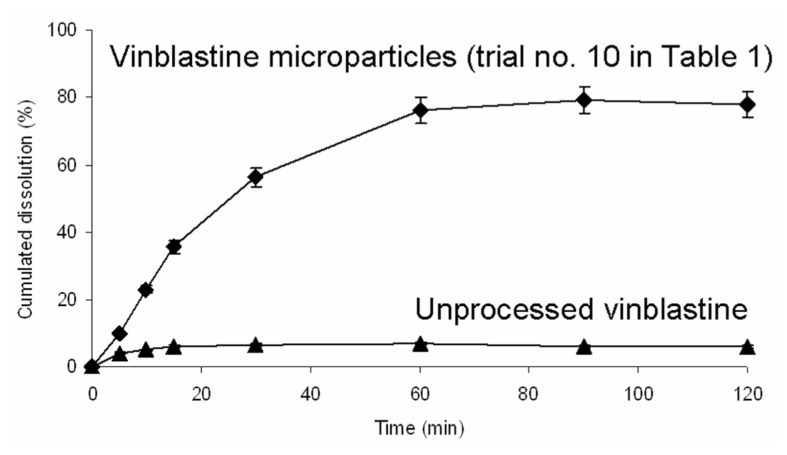
Dissolution profiles of the unprocessed and processed vinblastine in PBS.

The dissolution profiles of the raw vinblastine particles and vinblastine microparticles in dissolution medium (6.4 g Na_2_HPO_4_·12H_2_O; 0.6 g KH_2_PO_4_; 5.85 g NaCl in 1000 mL deionized water; pH 7.4) are shown in Figure 6. The vinblastine microparticles showed higher dissolution rate than the vinblastine raw particle. After 120 min, the dissolution rate of vinblastine microparticles and unprocessed vinblastine were 79.20% and 6.81%, respectively. The vinblastine microparticles had solubility eleven times higher than the unprocessed vinblastine material. The remarkable increase in the dissolution rate can be attributed to amorphous nature. The high internal energy and specific volume of the amorphous state relative to the crystalline state can lead to enhanced dissolution [15,17,18].

## 3. Experimental Section

### 3.1. Materials

Vinblastine was obtained from Zhejiang Hisun Pharmaceutical Co., Ltd. (Zhejiang, China). Carbon dioxide with high purity of 99.99% was supplied from Liming Gas Company of Harbin (Heilongjiang, China). *N*-Methyl-2-pyrrolidone (NMP) was purchased from Sigma (St. Louis, MO, USA). Deionized water was prepared by a Milli-Q water purification system (Millipore, Bedford, MA, USA) and was used in all experiments.

### 3.2. Apparatus

The schematic diagram of the supercritical antisolvent apparatus is illustrated in Zhao *et al.* [14]. The apparatus consists of a precipitation chamber and a gas-liquid separation chamber. The CO_2_ is cooled with a cooler before being compressed by a liquid pump and the pressure is controlled by a back pressure regulator. Afterwards, the CO_2_ is pre-heated in a heat exchanger and enters into the precipitation chamber. Simultaneously, the solution is pumped, heated and fed to the precipitation chamber through a stainless steel nozzle. This nozzle is located in a distinct inlet point from the CO_2_, but also in the top of the precipitation chamber. A stainless steel frit vessel was put into the precipitation chamber to collect the micronized particles and to let the SC–CO_2_/organic solvent mixture pass through. The flow rate of the mixture that leaves the precipitator is controlled by a valve located between the precipitation chamber and the gas-liquid separation chamber. Here the mixture undergoes a decompression to induce the separation of the CO_2_ from the organic solvent.

### 3.3. Supercritical Antisolvent Micronization

A supercritical antisolvent experiment begins by delivering supercritical CO_2_ to the precipitation chamber until the desired pressure is reached. CO_2_ steady flow of 8.5 kg/h is established; then, pure NMP is sent through the liquid pump to the precipitation chamber with the aim of obtaining steady state composition conditions during the vinblastine precipitation. At this point, the flow of the pure NMP is stopped and the vinblastine/NMP liquid solution is delivered through the nozzle of 150 μm. Once injected the fixed quantity of vinblastine/NMP solution, the liquid pump is stopped. After the spraying of drug solution into the particle precipitation vessel was completed, an additional supercritical CO_2_ continued to flow into the vessel at same rate for further 30 min to remove residual solvent from precipitated particles and to wash the stainless steel frit vessel of 200 nm from the residual content of liquid solubilized in the supercritical antisolvent. The precipitation chamber was then depressurized gradually to atmospheric pressure. Finally, the samples of micronized vinblastine were taken from the stainless steel frit vessel for further characterization.

### 3.4. Optimization of Supercritical Antisolvent Process

An orthogonal design L_16_(4)^5^ was selected for optimization of operating condition of vinblastine micronization by supercritical antisolvent process. The supercritical antisolvent experiment was carried out with 4 factors and 4 levels, namely vinblastine concentration (1.25, 2.50, 3.75, 5.00 mg/mL), precipitation temperature (40, 50, 60, 70 °C), precipitation pressure (10, 15, 20, 25 MPa) and drug solution flow rate (3.3, 6.7, 10, 13.3 mL/min). The range of each facto level was based on the results of preliminary experiments. The MPS (nm) of micronized vinblastine was the dependent variable. The data was analyzed using the Design Expert 8.0 software.

### 3.5. Powder Characterization

The MPS of processed samples was measured with DLS (ZetaPALS; Brookhaven Instruments Corporation, Holtsville, NY, USA) particle size analyzer. Processed vinblastine was suspended in filtered pure water and treated by sonication for 3 min to avoid the aggregation of particles. The whole process operated on a clean workbench to prevent dust or other particulate transmission. The water was presaturated with vinblastine to avoid dissolution of the micronized particles. The suspensions were analyzed in the DLS particle size analyzer. Every measurement was repeated three times. The MPS and standard deviations SD obtained were used to fit the particle size distribution to a lognormal distribution.

The morphology of particles was examined by SEM. Samples were coated with gold and palladium using a vacuum evaporator and examined using a SEM at 20 kV accelerating voltage. Analysis of the residual solvents was carried out on a (Quanta 200, FEI).

The samples were diluted with KBr mixing powder at 1% and pressed to obtain self-supporting disks, separately. The FTIR spectrum was obtained by MAGNA-IR560 E.S.P-1-1 (Nicolet, Madison, WI, USA) and recorded in the wave number range of 4000–500 cm^−1^ at a resolution of 4 cm^−1^.

The samples were dissolved in methanol, separately. MS spectra were obtained by analyst 1.4 of API 3000 (AB, Foster City, CA, USA). The mass spectrometer was operated in positive mode.

Diffraction patterns were recorded on a Rigaku Powder X-ray diffraction system, X-ray diffraction patterns were collected in transmission using an X-ray diffractometer with a rotating anode (Philips, Xpert-Pro, Almelo, The Netherlands) with Cu Kα1 radiation generated at 30 mA and 50 kV. The powders were filled to the same depth inside the sample holder by leveling with spatula and scanning rate (4 °/min) was same for all XRD analysis.

100 mg sample was added to a 500 mL dissolution medium (6.4 g Na_2_HPO_4_·12H_2_O; 0.6 g KH_2_PO_4_; 5.85 g NaCl in 1000 mL deionized water; pH 7.4). Bath temperature and paddle speed were set at 37 ± 0.5 °C and 100 rpm. At selected periods of 5, 10, 15, 30, 60, 90 and 120 min, 2 mL aliquots were withdrawn without media replacement, filtered, and analyzed using HPLC. The HPLC system consisted of a Waters 717 automatic sample handling system series HPLC system equipped with a 1525 Bin pump, a 717 automatic column temperature control box and a 2487 UV-detector (Waters, Milford, MA, USA). Chromatographic separation was performed on a Diamonsil C18 reversed-phase column (4.6 mm × 250 mm, 5 μm, Dikma Technologies). The quantification analysis method is according to Yang *et al*. [2]. The data was expressed as a mean value ± SD (*n* = 6). The percentage of cumulated dissolution was defined as the sum of the mass of dissolved vinblastine at time t, divided by the mass of added vinblastine (100 mg). The dissolution profiles were plotted as the percentage of cumulated dissolution *versus* incubation time.

## 4. Conclusions

In the present study, an orthogonal array design L_16_ (4)^5^ was used to determine the optimum experimental conditions for the supercritical antisolvent micronization of vinblastine from NMP. The result showed that the precipitation pressure has significant effect on the MPS of micronized vinblastine during the supercritical antisolvent process, whereas the other three factors showed some effect but insignificantly affected the result. The optimum micronization condition was at vinblastine concentration 2.50 mg/mL, precipitation temperature 60 °C, precipitation pressure 25 MPa and vinblastine solution flow rate of 6.7 mL/min. Moreover, that minimum MPS of vinblastine (121 ± 5.3 nm) was obtained. Moreover, the SEM, MS, FTIR and XRD allowed the characterization of micronized vinblastine. The results showed that the supercritical antisolvent process did not induce degradation of vinblastine and that processed vinblastine particles have lower crystallinity. Additionally, there is a great increase of dissolution rate in vinblastine microparticles by the supercritical antisolvent process compared to unprocessed vinblastine. In conclusion, the supercritical antisolvent process proposed in this study proved to be an efficient method for the preparation of vinblastine microparticles.

## Figures and Tables

**Table 1:** Orthogonal array design matrix L_16_ (4^5^) and experimental results.

Trial No.	A: Vinblastine concentration (mg/mL)	B: Drug solution flow rate (mL/min)	C: Precipitation pressure (MPa)	D: Precipitation temperature (ºC)	MPS (nm) (*n* = 3)
1	1.25	3.3	10	40	480
2	1.25	6.7	15	50	372
3	1.25	10.0	20	60	240
4	1.25	13.3	25	70	159
5	2.50	3.3	15	60	256
6	2.50	6.7	10	70	254
7	2.50	10.0	25	40	258
8	2.50	13.3	20	50	301
9	3.75	3.3	20	70	355
10	3.75	6.7	25	60	121
11	3.75	10.0	10	50	347
12	3.75	13.3	15	40	287
13	5.00	3.3	25	50	284
14	5.00	6.7	20	40	213
15	5.00	10.0	15	70	366
16	5.00	13.3	10	60	438

*K_1_*^a^	312.75	343.75	379.75	309.50	-
*K_2_*	267.25	240.00	320.25	326.00	-
*K_3_*	277.50	302.75	277.25	263.75	-
*K_4_*	325.25	296.25	205.50	283.50	-
*R*^b^	58.00	103.75	174.25	62.25	-

Optimal level	A_2_	B_2_	C_4_	D_3_	-

a *K_i_* = ∑(mean particle size at Ai)/4, the mean values of MPS for a certain factor at each level with standard deviation.

b R = max (K_i_) − min (Ki).
